# Recognition of Chiral Carboxylic Anions by Artificial Receptors

**DOI:** 10.3390/ijms11093334

**Published:** 2010-09-15

**Authors:** Pape Sylla Dieng, Claude Sirlin

**Affiliations:** Institut de Chimie UMR7177 CNRS, Université de Strasbourg, 4 Rue Blaise Pascal, 67070 Strasbourg, France; E-Mail: papedieng@chimie.u-strasbg.fr

**Keywords:** molecular recognition, anion recognition, chiral carboxylic anions recognition

## Abstract

Many carboxylic molecules, ranging from drugs to flavors and fragrances, contain chiral centers. As a consequence, research has been carried out in order to design and synthesize artificial receptors for carboxylic anions. Many problems have to be solved for binding anions. The results obtained in the binding of carboxylic anions by guanidine, secondary ammonium and metal-center have been selected. The last part of this review focuses on chiral recognition of carboxylic anions by organic and metal-based chiral receptors.

## 1. Introduction

Anions are ubiquitous in the natural world. Chloride is present in large amounts in the oceans; nitrate and sulfate are found in acid rain; carbonates are the key constituents of mineral materials. Anions are also critical for maintenance of life; they are involved in recognition, transport and transformation in almost every biochemical operation. For instance, anions are present in roughly 70% of all enzymatic sites, play structural roles in many proteins, and are critical for the storage of genetic information (DNA and RNA are poly-anions). Anions are also involved in activating signal transduction pathways, maintaining cell volume, and in the production of electric signals.

**Figure 1:**
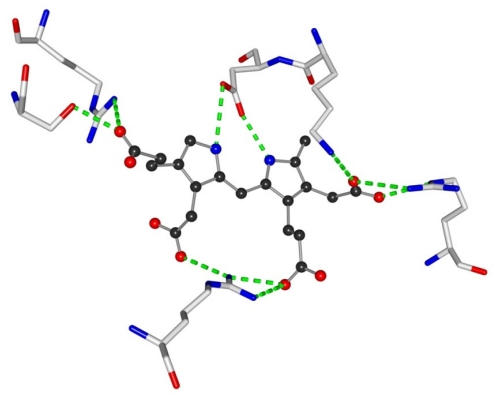
Partial view of the X-ray structure of the enzyme porpho-bilinogen de-aminase complex showing the binding of the di-pyrromethane co-factor through pyrrolic NH to Asp 84 and carboxylic side chains with Arg 155 and Lys 83.

X-ray structures have been solved allowing the direct visualization of the complexes between an enzyme and its anionic substrate, the complex being stabilized by a set of multiple hydrogen-ionic bonds. Especially indicative is the structure of the enzyme porpho-bilinogen de-aminase di-pyrromethane cofactor. The binding of the cofactor is ensured through the pyrrolic NH interactions with the oxygen atoms of the carboxyl side chain of Asp 84 (2.76 Ǻ and 2.81 Ǻ) and the dipyrromethane carboxylic interactions to Arg 155 (2.94 Ǻ and 3.01 Ǻ) and Lys 83 (2.89 Ǻ) [1] (see Figure 1).

Native zinc enzymes, such as carboxy-peptidase A, bind small inorganic anion like phosphate, chloride, azide, by a combination of metal-anion interaction and hydrogen bonding [2]. These hydrogen bonds originate from the peptide chain and from the water molecules bound in the active site.

The phenomenon of recognition is a central part of biology. It has not left chemists indifferent, and much research was carried out in order to design and synthesize artificial receptors. Molecular recognition is based on the principle of complementarities. The receptor and the substrate must display (i) geometrical complementarities, an important feature to ascertain the mutual stickiness of the binding partner (ii) electronic complementarities through positive/negative charge/dipole or hydrogen interactions. A sufficient binding force between the host and the guest must exist [3]. These principles are now applied in bottom-up fabrication of molecular self-assembly leading to an important concept in nanotechnology [4]. The design of receptors directed toward cations emerged first; anion recognition by itself is particularly challenging in comparison.

**Figure 2:**
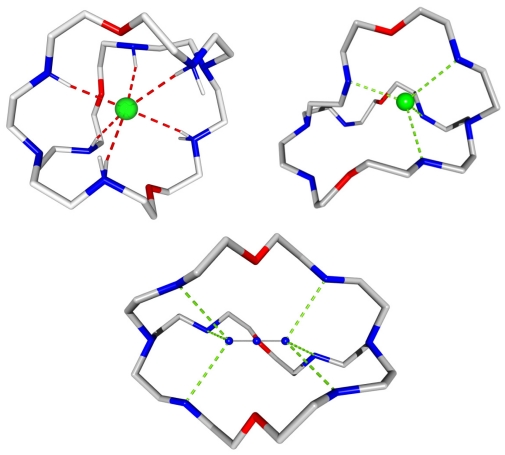
Depiction of the binding of chloride (left), fluoride (right) and azide (bottom) anions by the hexaprotonated macrobicyclic receptor bis-tren-6H^+^. Anions are encapsulated by the receptor through secondary ammonium hydrogen bonds with shape-dependant affinity.

Several problems need to be overcome: (i) anions are larger than their iso-electronic cations and have a lower charge to radius ratio (ii) they are stronger solvated; energy has to be paid to de-solvate the anion before its binding and therefore the stability of the anion complex will be smaller than that of the cationic species (iii) a variety of shapes has to be considered (iv) anions are susceptible to proton transfer and binding will only be effective in a definite pH window. As a consequence the binding site must be a single or multiple Lewis acid, cationic or electron-deficient, to complement the negative charges of the anionic substrate [5–7]. X-ray structures are presented where these requirements have been more or less fulfilled in the complexed species (Figure 2) [8]. As the binding of chloride and azide anions by the hexa-protonated macro-bicyclic receptor bis-tren-6H^+^ appears to satisfy the concept of structural complementarities, the X-ray structure of the fluoride complex displays large distortion of the ligand in the complex. Anions are encapsulated by the receptor through the secondary ammonium hydrogen bonds with a shape-dependant affinity.

Among the anions, carboxylic anion is a particularly common functional group and has inspired the development of a number of different approaches for its recognition. Indeed, carboxylic anions are involved in several molecular recognition phenomena of biological interest. Carboxylic acids are present in amino acids, enzymes, antibodies and metabolic intermediates where they contribute to their characteristic biochemical behavior. Also, hydrogen-bonding arrangements, involving one or both oxygen atoms of carboxylic acid, take an important part in non-covalent organization of secondary and tertiary structures of complex biological molecules.

## 2. Recognition of Carboxylic Anions by Guanidine Based Receptors

**Figure 3:**
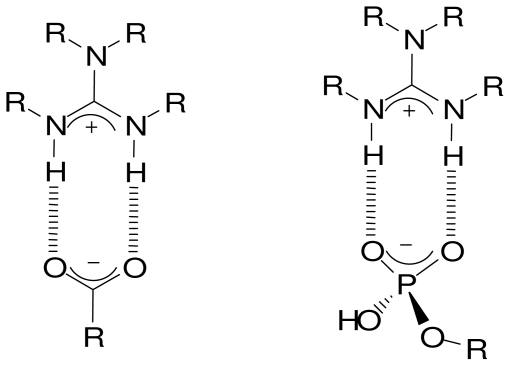
Binding pattern of the guanidine moiety toward carboxylic and phosphate anions deduced from a guanidine-nitrate complex X-ray structure.

The guanidine unit is present in the arginine side chains and is involved in the binding and recognition of ionic substrates, in addition its important function in maintaining protein tertiary structure. The reason for the strong interaction, especially with the oxy-anions, lies in the binding pattern featuring two parallel hydrogen bonds in addition to the electrostatic attraction (Figure 3). Otherwise, the guanidine moiety is an attractive group of artificial receptors due to its low acidity (pK = 13.5) which maintains its cationic state in a wide pH range. The X-ray structure of a guanidine-nitrate complex has been solved [9]. As an analogy, a carboxylic and phosphate complex could be proposed.

The synthesis of guanidine containing macro-cycles has also been achieved. Selectivity revealed that carboxylic anions are stronger bound than halides [10,11]. Later, bifunctional cyclic guanidine containing hosts, were developed and displayed affinity and selectivity for a wide variety of di-carboxylic anions in methanol (malonate is bound with pK = 4.2 and isophtalate with pK = 3.7) [12] Improved binding came from more pre-organized hosts. Indeed the guanidine group is embedded in a bicyclic framework that could reduce hydration of the charged moiety by the accumulation of hydrophobic hydrocarbon residues as well as lead to the predictability of the host-guest orientation.

Urea and thio-urea are frequently employed as anion binding sites in neutral organic anion receptor species. Ureas form chelated complexes with di-topic hydrogen bond acceptors such as phosphate, sulphuric and carboxylic acids [13].

## 3. Recognition of Carboxylic Anions by Secondary Ammonium-Based Receptors

**Figure 4:**
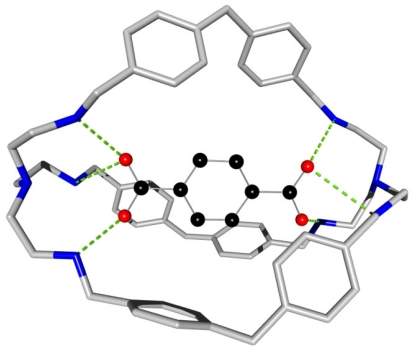
Binding of the terephtalate anion to the tris (biphenyl) crytand through secondary ammonium hydrogen bonds and additional arene interactions.

A fair success on the use of the guanidinium unit prompted research on protonated macrocyclic amines. The triprotonated [14] form of the N_6_ [18] ane and the octaprotonated [15] N_8_ [32] ane mostly bind their guests by electrostatic interactions, the binding constant becoming higher as the number of protonated nitrogen atoms increases. As exemplified, the binding selectivity of the receptors presents a striking chain length dependence of the binding of the dicarboxylates guests: the N_6_ [32] ane-6H^+^ binds the glutarate anion (pK = 4.4) more strongly, while pimelic anion is better recognized by the N_6_ [38] ane-6H^+^ (pK = 4.4) in aqueous solutions [16]. The crystal structure of the complex formed between a cyclophane-type macrobicycle and the terephtalate anion shows a good receptor/substrate through optimal contact and complementarities, emphasized by the additional arene-arene interactions (Figure 4) [17].

Macrotricyclic quaternary ammonium salts were demonstrated to bind carboxylic anions, such as formate, acetate, and benzoate [18]. Although these receptors do not display hydrogen bond interactions, they feature well-localized polycationic centers independent of the acidity of the medium. Complex stability increases with increasing charge of the anion, in the order CO_3_^2−^ > HCO_3_^−^, or HPO_4_^2−^ > H_2_PO_4_^−^, consistent with a binding mechanism that is based on electrostatic interactions.

## 4. Recognition of Carboxylic Anions by Metal Based Receptors

A metal center can contribute directly to anion binding, either by using its positive charge to attract the anion by electrostatic interaction and/or by acting as Lewis acidic binding site. Metal complexes play an important role in anion chemistry due to the possibility of introducing a range of advantageous physicochemical properties. In the majority of cases, the metal complex is incorporated as a reporter group whose photochemical or red-ox response is changed upon proximal binding of an anion. The metal complex motif can also be utilized as a structural component in anion receptors. By exploiting these different properties, often in combination, metal complex anion receptors achieve a range of functionality beyond the scope of purely organic based receptors.

**Scheme 1:**
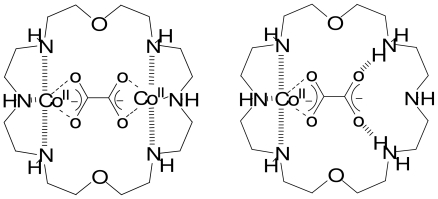
Oxalate anion binding to cobalt centers by a macro-cyclic receptor.

A possibility in the design of carboxylic anions receptors is the incorporation of a metal center with which the carboxylic anion may form a direct coordination bond. Early results in this area come from the description of oxalate inclusion in a cobalt (II) complex (Scheme 1) [19,20].

**Figure 5:**
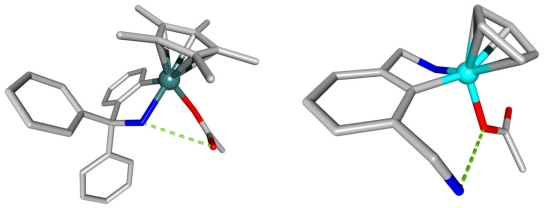
X-ray structures of two different acetate complexes. Left: an iridium (III) complex where the metal binds the carboxyl site, the carbonyl oxygen displaying H-bond with the NH function of the ligand. Right: a cobalt (III) complex where the metal binds the carboxyl site and displays simultaneously H-bond interaction with the NH function of the ligand.

A cobalt-organic compound was shown to bind various anions such as acetate with the combination of coordination to the cobalt atom and a hydrogen-bond (2.724 Ǻ). This receptor demonstrated to be efficient in a slightly acidic solution. Higher pH water molecule coordinates, through a hydrogen bond to the dimethylamine unit by the host and the water molecule, inhibits the anion binding. Selectivity towards the acetate and phosphate anions (pK = 3.1) was observed compared to chloride (pK = 1.8) [21,22]. The crystal structure showed that the substrates are bound through a seven-member cycle (the carboxyl group binds directly to the cobalt center and a hydrogen bond is displayed with the dimethylammonium unit of the ligand (Figure 5).

Later a pseudo-tetrahedral iridium complex was isolated with acetate as a coordinated ligand. The structure of the acetate complex determined by X-ray crystallography and NMR spectroscopy revealed that there is a short N-O distance of 2.945 Ǻ, indicating an intramolecular hydrogen bond (Figure 5) [23].

Anion receptors incorporating the red-ox active cobaltocene group have been studied extensively due to the combination of an accessible red-ox couple and favorable electrostatic interactions of the cationic metallocene motif with anions [24]. Electrochemical sensing anion was demonstrated with such systems. The high sensitivity of fluorescent techniques for sensing anions species has created an enormous interest in the field of anion recognition. Among the examples of luminescent anion-responsive systems, the tris (2,2′-bipyridyl)ruthenium(II) ([Ru(bpy)_3_^+^]) is the most intensively investigated. Luminescence measurements were undertaken to probe the anion binding process [25,26].

**Figure 6:**
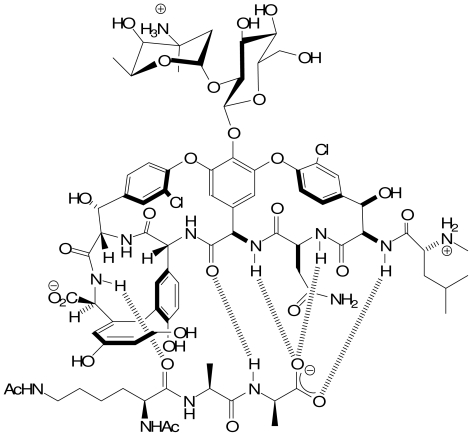
Schematic representation of the interactions displayed between vancomycin and the peptide Ac-l-Lys-(Ac)-d-Ala-d-Ala.

The enantio-selective recognition of chiral carboxylic anions is an important goal because numerous pharmaceutical compounds possess this functional group. It is worth noting the special role of carboxylic acid recognition in determining the biological activity of the vancomycin family of antibiotics [27]. Vancomycin and related glyco-peptides activities are due to the inhibitory effect of these compounds on the cross-linking of peptido-glycan precursors involved in bacterial cell wall biosynthesis [28,29] The antibiotic blocks the action of the bacteria by binding the d-Ala-d-Ala dipeptide. The crystal structure of the complex between vancomycin and the peptide Ac-l-Lys-(Ac)-d-Ala- d-Ala shows three intra-molecular hydrogen bonds between the vancomycin NH groups and the terminal acid of the peptide (Figure 6) [30–32]. It was shown that the main contribution to complex stability comes from the interactions involving the terminal acid [33].

**Figure 7:**
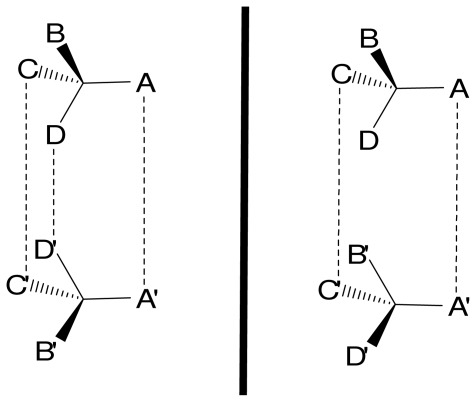
The three-point interaction model. With one enantiomer three interactions are established, but only two are possible with the other enantiomer. The third interaction D-D′ determines the degree of enantio-selectivity.

The design and synthesis of enantio-selective host molecules carry a great importance for synthetic and analytical purposes. Chiral recognition implies and includes the formation of two diastereomeric species between the chiral receptor and the enantiomeric substrates. The energetics of the process lies on the different stabilities of the two resulting complexes. Enantio-discrimination in host-guest systems has previously been reviewed [34]. The treatment of the chiral information is under the control of the three-point rule [35–38]. That rule governs the design of enantio-selective receptors; three simultaneous interactions between the receptor and one of the enantiomers must be established with at least two stereo-specific interactions (Figure 7). It should be stressed that some modifications of this model have been discussed, but in general this model applied to most of the chiral separation principles [39].

For the least preferred enantiomer, binding with the receptor is weaker due to a less favorable orientation. The equilibrium constant for the complex formation is therefore smaller than that for the preferred enantiomer. The ratio of the equilibrium constants is a quantitative measure of the intrinsic selectivity of the receptor.

## 5. Recognition of Chiral Carboxylic Anions by Organic Receptors

**Scheme 2:**
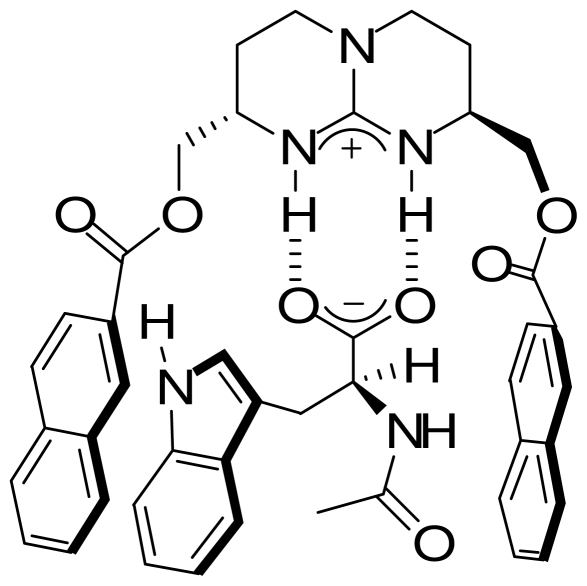
*N*-acetyl-tryptophan binding to a chiral guanidine receptor.

Chiral recognition of anions has previously been reviewed [40]. The first result was obtained with the carboxylic enantio-selective recognition through a chiral bicyclic guanidine receptor. The chirality of the receptor forces any substrate to assume a favorable conformation in a dissymmetric environment. Extraction experiments of the l- and d-tryptophan derivatives with both enantiomers of the receptor (*SS)* and (*RR*) afforded the corresponding diastereomeric salts (de = 17%). Stacking interactions with the aromatic part of the receptor, revealed by complexation-induced shifts in the NMR spectra, account for the selectivity observed for aromatic over aliphatic carboxylic acids (Scheme 2) [41].

**Scheme 3:**
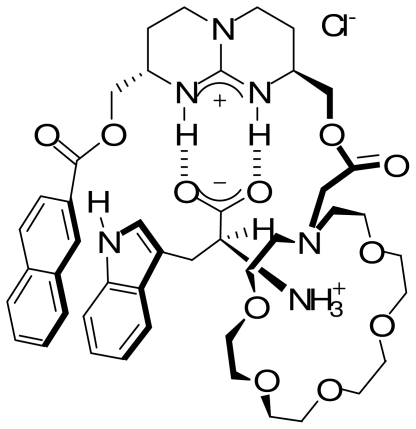
Trytophan zwitter-ion binding to a crown-guanidine conjugate.

Furthermore a bicyclic guanidine-based receptor, having a crown ether anchor group on one side, and an aromatic naphthalene ester on the other, was shown to bind zwitterionic amino acids. Extractions selectivities were obtained for aromatic amino acids supporting a three-point host-guest interaction mode [42]. For instance l-Leu-l-Trp was extracted with a de of 99% (Scheme 3).

**Scheme 4:**
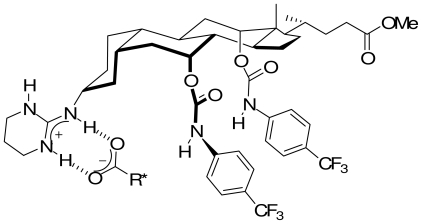
Carboxylic anion binding to a guanidine-steroid conjugate.

Receptors bearing a guanidine unit anchored to a steroidal framework were shown to achieve good chiral extraction of *N*-acetyl alanine/phenylalanine carboxylic anions with L: D ratios of 7–10:1 [43]. Moreover a lipophilic steroidal analogue demonstrated its effectiveness as an enantioselective carrier for *N*-acetyl-α-amino carboxylic anions through bulk liquid membranes (Scheme 4). Selectivity up to 70% ee was obtained [44].

Urea has shown to provide a good binding site for carboxylic anions through the bidirectional hydrogen-bonding motif. A cyclic bis chromenylurea bridged by amide functions as H-bond donors was achieved. High chiral recognition for naproxenate was reported in a competitive experiment between a racemic mixture of the receptor and the (*S*)-naproxen salt [45].

**Scheme 5:**
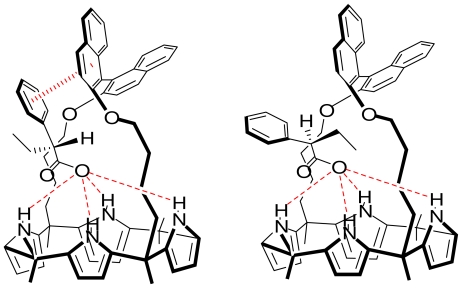
Carboxylic anion binding to a binol strapped porphyrin.

Among the various neutral anion-binding agents reported to date, calixarene [4] pyrrole has attracted particular attention because it binds carboxylic acids. A binol-strapped calixarene [4] pyrrole was synthesized and was able to recognize and bind appropriately 10-times larger the (*S*)-phenyl-butyrate substrate than its (*R*) enantiomer in CD_3_CN. Besides the pyrrole NH-carboxylic hydrogen bonds, the selectivity was rationalized in terms of favorable arene-arene interactions between the naphtyl part of the receptor and the phenyl substrate unit [46] (Scheme 5).

High enantio-selection of α-hydroxy-carboxylic acids has been achieved with 1,1′-bis-2-naphtol-amino alcohol molecules. Based on fluorescence measurements, phenyl-lactic or hexahydro-mandelic acids enantiomers were discriminated with a fluorescence intensity ratio of 26 [47].

## 6. Recognition of Chiral Carboxylic Anions by Metal-Based Receptors

**Scheme 6:**
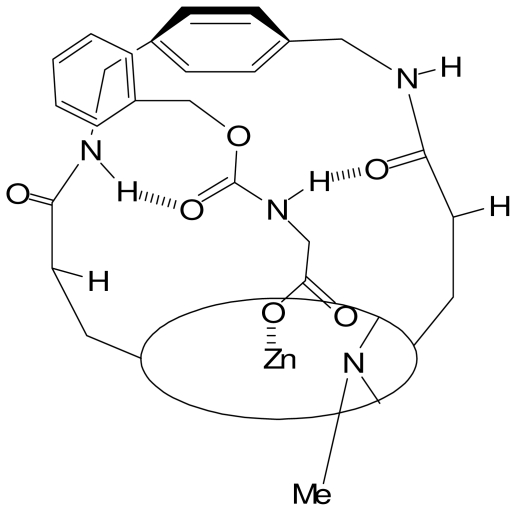
*N*-carbamate-glycine anion binding to a zinc-porphyrine.

Designing chiral metal complexes for enantio-selective recognition of carboxylic acids has been achieved for the first time with Zn-porphyrine. A chiral *N*-methylated porphyrin, where the methyl group blocks an approach from the lower face, demonstrated to bind selectively amino acid in the chiral environment provided by the strap. Amino acids were extracted from water with diastereomeric excesses reaching 90% [48]. Investigation using IR and ^1^H NMR suggested that binding involved an electrostatic zinc-anion interaction and additional hydrogen bonds (Scheme 6).

A hydrophobic chiral salen-cobalt (III) complex was also developed and achieved a resolution of racemic *N*-benzyl α-amino carboxylic acids in excellent enantiomeric excesses reaching 93%. The substrate could be released by reduction of the Co (III) to Co (II) center followed by counter-extraction, regenerating the properties of the carrier [49].

**Figure 8:**
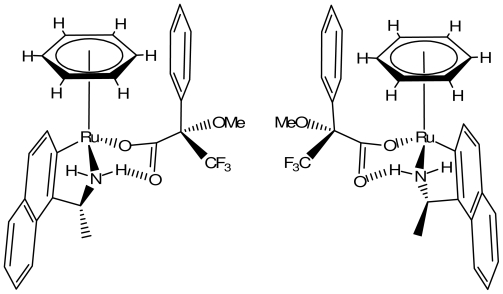
(*RS_Ru_*),(*R*) (right side) and (*RR_Ru_*),(*S*) substrate-receptor structures: the carboxylic function interacts with the Ru^+^-NH unit; CH interacts with π system.

Tetrahedral half-sandwich transition-metal complexes offer the possibility to build in a chiral metal center. As a consequence, they represent an interesting means for the preparation of chiral discriminating agents. Cyclo-metallation of a chiral ligand induces chirality at the metal center. Related tetrahedral ruthenium [50,51] and cobalt [52] compounds have been described. They open routes towards the development of chiral carboxylates metal-organic receptors by combining the functional properties of a chiral metal center with the recognition capabilities of hydrogen bonding groups. A chiral tetrahedral ruthenium compound was demonstrated to complex and discriminate two enantiomeric carboxylate substrates. Complexation was highlighted by spectra changes observed in solution either by NMR or IR. A selectivity factor of 6 was measured in an extraction experiment [53]. This selectivity is in the order of magnitude of values generally found in the literature, but with quite different receptor systems. Due to configurationally labile metal-center, the two (*R*) and (*S*) substrates are bound, but each specifically to one stereo isomer of the receptor (Figure 8); an observation that may be related to induce fit phenomenon [54,55].

## 7. Conclusion

Chiral centers occur in many carboxylic molecules such as drugs, flavors and fragrances. As a consequence, the scientific and economic relevance of chiral substances in academic and industrial research is displayed, favoring the development of chiral organic and transition-metal receptors towards carboxylic anions. Application of the recognition phenomenon could be found mainly in analytical chemistry through the development of, for example, chromatography, liquid-liquid extraction and transport. It should be noted that simultaneously biotechnologies and biocatalysis are expanding in the production and purification of chiral molecules containing carboxylates.
